# Trends and predictors of cemented fixation in arthroplasty for patients with a hip fracture: a Canadian Joint Replacement Registry study

**DOI:** 10.2340/17453674.2025.44751

**Published:** 2025-10-27

**Authors:** Christiaan H RIGHOLT, Colton POITRAS, Gavin C A WOOD, Eric R BOHM

**Affiliations:** 1 Orthopaedic Innovation Centre, Winnipeg; 2 Department of Surgery, University of Manitoba, Winnipeg; 3 College of Pharmacy, University of Manitoba, Winnipeg; 4 Biomedical Engineering Program, University of Manitoba, Winnipeg; 5 Division of Orthopaedic Surgery, Queen’s University, Kingston; 6 Concordia Joint Replacement Group, Winnipeg, Canada

## Abstract

**Background and purpose:**

Cemented fixation is widely recommended during arthroplasty for hip fractures, yet it has not been universally adopted by all surgeons and hospitals. We aimed to identify which factors were associated with a higher likelihood of cemented fixation.

**Methods:**

We identified patients ≥ 55 years old with hip fractures treated with primary arthroplasty in Canada between 2012 and 2022. We determined fixation method along with several surgeon and hospital characteristics from the Canadian Joint Replacement Registry and other Canadian databases. We used logistic regression to estimate the odds ratios (ORs) of the association between these covariates and cemented fixation.

**Results:**

We identified 65,823 patients who underwent arthroplasty for hip fractures. The proportion of cases with cemented fixation for hemiarthroplasty increased by 30 percentage points over the study period; the proportion for total hip arthroplasty (THA) remained relatively stable. High-volume hospitals (≥ 500 annual hip arthroplasties with ≥ 25% of these for fractures) were least likely to cement, OR = 0.30; 95% confidence interval (CI) 0.27–0.34 vs low-volume hospitals (< 500 cases/< 25% for fractures). High volume surgeons (≥ 50 hips/year, ≥ 16/year for fractures) had an OR of 0.80 (CI 0.75–0.84) compared with < 50 hips/year surgeons. Teaching hospitals were more likely to cement, OR = 1.16 (CI 1.10–1.22). The OR of cemented fixation for hemiarthroplasty (vs THA) rose from 1.13 (CI 0.99–1.29) at the start of the study period to 2.17 (CI 2.02–2.33) at the end.

**Conclusion:**

The use of cemented fixation in hip fracture arthroplasty has increased across Canada over the last decade. However, surgeons and hospitals that treat more hip fracture patients are less likely to cement. The proportion of cemented fixation increases with patient age. Cement use is more common in hemiarthroplasty than in THA.

Cemented fixation is internationally recommended when using arthroplasty to treat hip fractures due to its superiority over cementless fixation [1-4]. There are many benefits of cemented fixation over cementless fixation, for example a lower risk of revision [5,6], a lower risk of periprosthetic fracture [7], better quality of life [7], and faster mobilization [8]. Some argue cement use increases the risk of mortality and morbidity due to bone cement implantation syndrome and its cardiopulmonary sequalae. However, there is no association between cemented fixation and cardiac injury assessed using elevated troponin [9], 10- or 30-day mortality [10], or 1-year mortality [11].

Despite the broad evidence base and widespread guidelines to cement arthroplasties for hip fractures, cemented fixation is not universally adopted; its prevalence is only 50% in the United States [12] and 58% in Canada [13]. This prevalence is higher elsewhere, e.g., 80% in England [14], 86% in Germany [15] and the Netherlands (for hemiarthroplasty) [16], and 98% in Sweden [17].

Little is known about what contributes to the choice of fixation, e.g., whether certain patient, surgeon, or hospital characteristics are associated with higher use of cemented fixation. To assess this, we built on our earlier work [13] and aimed to identify longitudinal trends by stratifying cement prevalence by these various potential predictors and by measuring whether any were associated with a higher likelihood of cemented fixation. The ultimate goal is to guide future quality improvement initiatives that can increase the uptake of cemented fixation.

## Methods

We conducted a registry study using several national databases housed and maintained by the Canadian Institute for Health Information (CIHI). We reported this study in accordance with the Strengthening the Reporting of Observational Studies in Epidemiology (STROBE) statement.

### Data sources

The Canadian Joint Replacement Registry (CJRR), in operation since 2001, records detailed information on hip and knee replacements across Canada and captures administrative, clinical, and prosthesis information. Submission to the CJRR is mandatory in 4 (of 10) provinces, containing about 60% of the Canadian population, and includes around 75% of all hip and knee arthroplasty Canada-wide [18] and 88% in the included regions (see below). Mandatory reporting started at different times in different provinces; > 50% of the Canadian population has been covered since April 1, 2012 [18]. CIHI is an interprovincial organization that receives data directly from Canada’s 10 provinces and 3 territories. Different databases are linkable using unique patient identifiers.

CIHI maintains records of inpatient hospital services in Canada since April 1994 in the Discharge Abstracts Database (DAD) [19]. Data is entered directly from acute care facilities upon discharge of the patient. There is mandatory reporting of inpatient services to the DAD across Canada, except for Quebec, and there is mandatory reporting of day surgery to the DAD in 4 provinces and all 3 territories. The DAD contains demographic, clinical, and administrative data; since April 2004 all patient diagnoses are coded using the International Classification of Disease, Tenth Revision, Canada (ICD-10-CA) and all procedures are coded using the Canadian Classification of Health Interventions (CCI). The National Ambulatory Care Reporting System (NACRS) has collected information on ambulatory care since April 2001 and has exclusively used ICD-10-CA and CCI coding since April 2004 [20]. NACRS contains information collected at the time of service on day surgery (mandated in 5 provinces), outpatient and community clinics, and emergency departments (mandated in 3 provinces and 1 territory). Day surgery in Quebec is reported to neither the DAD nor NACRS. Since April 2018, we obtained a unique surgeon identifier from DAD and NACRS, and used the patient-level physician billing database (PLPB) for the 3 provinces where a unique provider number was not available.

### Study cohort

Our study cohort consisted of every patient 55 years of age or older with provincial health insurance coverage who had primary hemiarthroplasty or total hip arthroplasty (THA) in 1 of 9 Canadian provinces (all provinces except Quebec) for an acute hip fracture between April 1, 2012 and March 31, 2022 (the study period) and whose age and sex were known. We excluded patients who had surgery in Quebec, due to incomplete entry into the CJRR in that province, or in the territories, due to the small number of procedures done there. We identified primary surgeries from the DAD/NACRS with the appropriate 1.VA.53 CCI procedure subcode for hemiarthroplasty (PM as digit 8–9) or THA (PN as digit 8–9) using either the anterior (LL as digit 6–7) or lateral/posterior (LA as digit 6–7) approach and ICD-10-CA code S72 as most responsible diagnosis, with digit 4–6 identifying the location of the fracture in the femur.

### Outcome and covariates

We identified the use of cement for fixation from the presences of cement product information in the CJRR and the 10th digit of the arthroplasty CCI code, which is based on the presence of cement stickers and coding of the chart, respectively. While it is not possible to know precisely which component is cemented, the use of reverse hybrid THA in Canada is rare and therefore it is safe to assume that the fixation method applied to the femoral component. We obtained age, sex, province, and date of surgery from DAD/NACRS. We defined the wait time as the time from admission to time of arthroplasty and identified comorbidity using the Charlson comorbidity index definitions validated for Canadian data [21]. The hospital was classified as a teaching or community hospital as defined by its submitting province’s definition. We classified annual hospital volume as low (< 500 hip arthroplasties/year) or high (≥ 500 hip arthroplasties/year) and annual hip fracture volume as the proportion of hip arthroplasties for hip fractures (under/over 25%). We classified surgeons as low volume if they had an annual volume of < 50 hip arthroplasties/year and grouped the high-volume hip surgeons, ≥ 50 hip arthroplasties/year, as under/over 16 hip fracture arthroplasties/year (approximately the median at the patient level).

### Statistics

Our study should be seen as a hypothesis-generating study to identify factors that may be associated with reduced use of cementing as possible elements on which to focus quality improvement efforts. To do this, we first plotted the proportion of procedures using cemented fixation over time separately for hemiarthroplasty and THA, because of the difference in cementing prevalence between the 2 types of surgeries. We then used logistic regression to estimate the odds ratios (ORs) and 95% confidence intervals (CIs) of the association between various demographic and clinical characteristics and cemented fixation while mutually adjusting for the other characteristics and stratifying by type of arthroplasty. Because of the differences over time and the limited period for which surgeon information was available, we stratified this analysis by year of surgery. We used the regression for the latest period (2019/20–2021/22) to estimate the marginal distribution (“adjusted proportion”) of cemented fixation by age, accounting for all other characteristics. We repeated this analysis for each province for 2021/22 and used random effects meta-analysis to compute an overall meta-estimate and assessed the interprovincial heterogeneity of each measure using the I^2^ and τ^2^.

We used Stata 18 (StataCorp, College Station, TX, USA) for all analysis.

### Ethics, data sharing plan, funding, and disclosures

This study was approved by the University of Manitoba Research Ethics Board (HS25086 (H2021:275)). The original source data is not owned by the researchers or CIHI and as such cannot be provided to a public repository. This study was funded by the Concordia Foundation and the University of Manitoba’s Alexander Gibson Research Fund. The opinions presented in the report do not necessarily reflect those of the funders. Authors state no conflict of interests. Complete disclosure of interest forms according to ICMJE are available on the article page, doi: 10.2340/17453674.2025.44751

## Results

**Figure 1:**
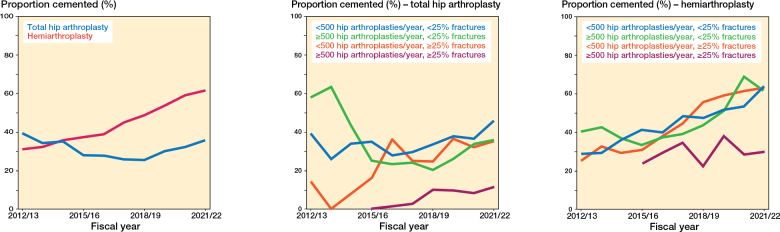
Trend in the proportion of hip fracture patients with cemented fixation (%) by type of arthroplasty.

**Figure S1:**
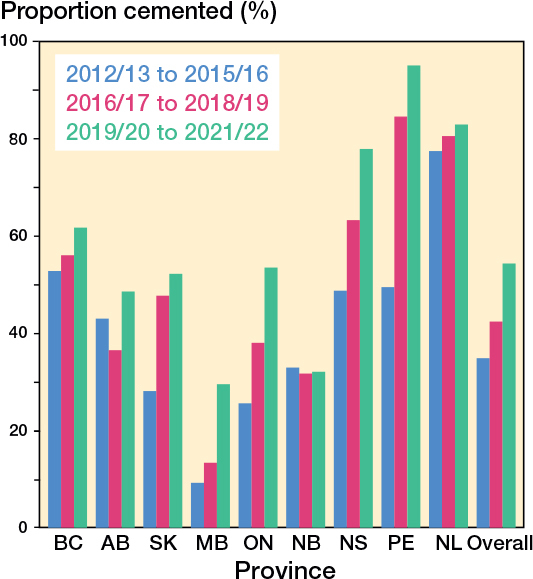
Cementing proportion (%) by province and period.

We included 65,823 patients, around two-thirds of whom were female and over 60% of whom were 80 years of age or older (Table 1). The median age of surgery was youngest for cementless THA, at 72 years old, and highest for hemiarthroplasty, at 84 years old for both cementless and cemented hemiarthroplasty, and the vast majority of fractures, > 97%, were femoral neck fractures. Comorbidity was more prevalent in groups with higher ages (Table 1, Table S1, see Appendix). The proportion of hemiarthroplasty cases with cemented fixation increased by about 30 percentage points from 32% in 2012/13 to 63% in 2021/22, whereas the proportion cemented for THA dipped from 39% in 2012/13 to 26% in 2018/19 and rebounded to approximately the same level by 2021/22 (Figure 1). The proportion of patients with cemented fixation increased in all but 1 province, New Brunswick, while increasing overall from 32% to 57% over the 10-year study period (Figure S1, Table S2, see Appendix).

**Table 1:** Hip fracture patients 55 years and older by fixation and type of arthroplasty. Values are count (%) unless otherwise specified

Factor	Total hip arthroplasty		Hemiarthroplasty	
	Cementless n = 6,338	Cemented n = 2,791	Cementless n = 28,850	Cemented n = 27,844
Sex				
Female	3,992 (63)	1,973 (71)	19,288 (67)	19,309 (69)
Male	2,346 (37)	818 (29)	9,562 (33)	8,535 (31)
Age at surgery				
55–64	1,557 (25)	319 (11)	1,369 (4.7)	986 (3.5)
65–79	3,010 (47)	1,004 (36)	8,446 (29)	7,628 (27)
≥ 80	1,771 (28)	1,468 (53)	19,035 (66)	19,230 (69)
Age at surgery, median (IQR)	72 (65–81)	80 (71–87)	84 (76–89)	84 (78–90)
Comorbidity	827 (13)	572 (20)	6,760 (23)	6,532 (23)
Province				
British Columbia	988 (16)	623 (22)	3,900 (14)	6,127 (22)
Alberta	1,111 (18)	355 (13)	3,444 (12)	3,147 (11)
Saskatchewan	205 (3.2)	33 (1.2)	1,552 (5.4)	1,419 (5.1)
Manitoba	473 (7.5)	47 (1.7)	2,131 (7.4)	615 (2.2)
Ontario	3,188 (50)	1,248 (45)	15,063 (52)	12,438 (45)
New Brunswick	162 (2.6)	152 (5.4)	1,603 (5.6)	681 (2.4)
Nova Scotia	135 (2.1)	92 (3.3)	777 (2.7)	1,803 (6.5)
Prince Edward Island	17 (0.3)	89 (3.2)	103 (0.4)	327 (1.2)
Newfoundland & Labrador	59 (0.9)	152 (5.4)	277 (1.0)	1,287 (4.6)
Period				
2012/13–2015/16	810 (13)	373 (13)	7,065 (24)	3,833 (14)
2016/17–2018/19	2,268 (36)	810 (29)	10,951 (38)	8,897 (32)
2019/20–2021/22	3,260 (51)	1,608 (58)	10,834 (38)	15,114 (54)
Hospital type				
Community hospital	3,878 (61)	1,756 (63)	19,431 (67)	17,538 (63)
Teaching hospital	2,460 (39)	1,035 (37)	9,419 (33)	10,306 (37)
Annual hospital hip arthroplasty volume				
< 500 (< 25% fracture)	2,016 (32)	1,078 (39)	11,490 (40)	9,459 (34)
< 500 (≥ 25% fracture)	1,962 (31)	929 (33)	9,825 (34)	12,381 (44)
≥ 500 (< 25% fracture)	1,944 (31)	745 (27)	6,217 (22)	5,399 (19)
≥ 500 (≥ 25% fracture)	416 (6.6)	39 (1.4)	1,318 (4.6)	605 (2.2)
Annual surgeon hip arthroplasty volume				
< 50	1,073 (17)	784 (28)	6,785 (24)	9,413 (34)
≥ 50 (<16 fractures)	1,037 (16)	498 (18)	2,893 (10)	3,717 (13)
≥ 50 (≥16 fractures)	1,945 (31)	591 (21)	4,599 (16)	5,053 (18)
Missing	2,283 (36)	918 (33)	14,573 (51)	9,661 (35)
Approach				
Lateral/posterior	5,590 (88)	2,388 (86)	26,797 (93)	26,316 (95)
Anterior	748 (12)	403 (14)	2,053 (7.1)	1,528 (5.5)
Fracture location				
Femoral neck	6,102 (96)	2,643 (95)	28,340 (98)	27,227 (98)
Intertrochanteric area	206 (3.3)	129 (4.6)	386 (1.3)	535 (1.9)
Subtrochanteric area	30 (0.5)	19 (0.7)	124 (0.4)	82 (0.3)
Wait time (admission to surgery)				
≤ 24 hours	3,642 (57)	1,400 (50)	15,631 (54)	13,814 (50)
> 24 hours	2,696 (43)	1,391 (50)	13,219 (46)	14,030 (50)

Comorbidity is any of the comorbidities included in the Charlson comorbidity index [21], §see Table S1 for individual comorbidities; IQR: interquartile range.

**Figure 2:**
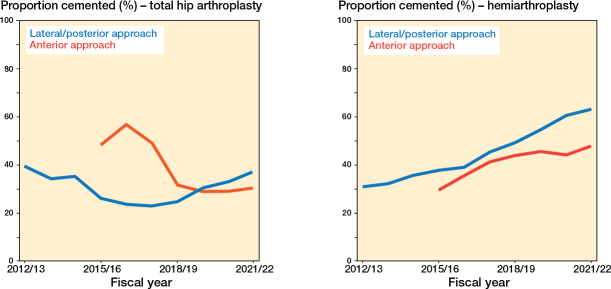
Trends in the proportion of hip fracture patients with cemented fixation (%) by annual hospital hip arthroplasty volume, approach, and type of arthroplasty.

**Figure S2:**
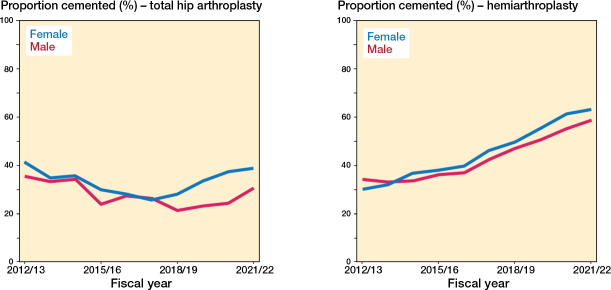
Trends of the proportion of hip fracture patients with cemented fixation (%) by sex and type of arthroplasty.

**Figure S3:**
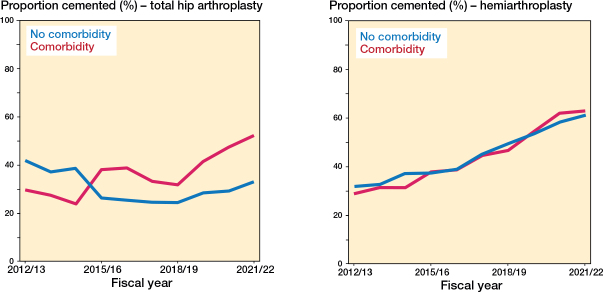
Trends of the proportion of hip fracture patients with cemented fixation (%) by comorbidity and type of arthroplasty.

**Figure S4:**
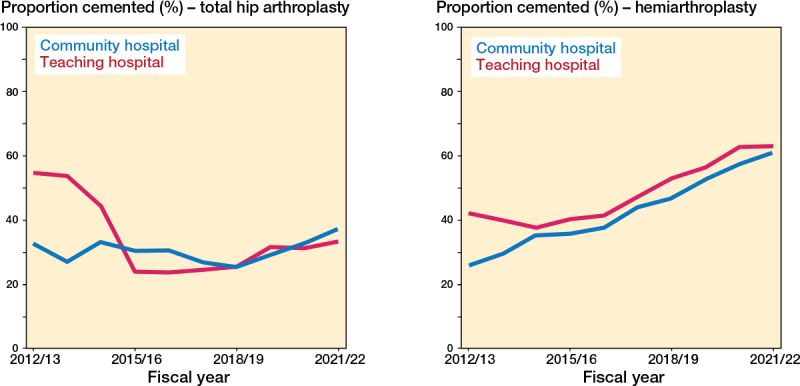
Trends of the proportion of hip fracture patients with cemented fixation (%) by hospital type and type of arthroplasty.

**Figure S5:**
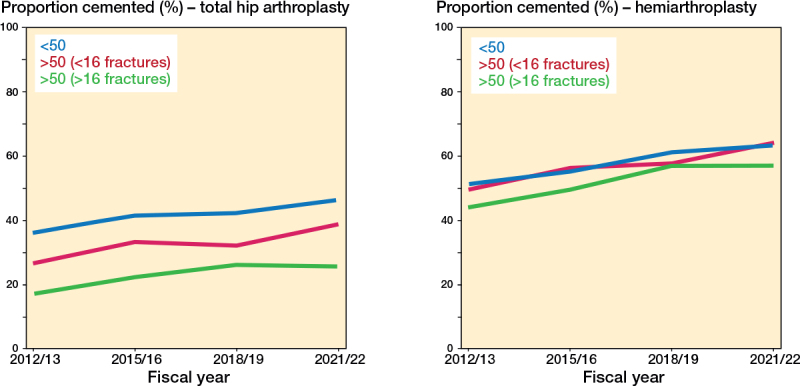
Trends of the proportion of hip fracture patients with cemented fixation (%) by surgeon category and type of arthroplasty.

The proportion of cemented cases increased for each hospital group over time, except for high-volume hospitals with a high proportion of fractures (Figure 2). In 2021/22, operations through the anterior approach were less likely to include cemented fixation than those with lateral/posterior approaches (Figure 2). Both high-volume high-fracture hospitals and anterior approach surgery represent a relatively low proportion (< 10% each) of the overall patient population (see Table 1). Male patients receive cemented implants less frequently (Figure S2, see Appendix) as do THA patients without comorbidity (Figure S3, see Appendix). Teaching hospitals seem to consistently lead community hospitals in cemented fixation for hemiarthroplasty (Figure S4, see Appendix), while high-volume surgeons with ≥ 16 hips/year for fractures are less likely to use bone cement (Figure S5, see Appendix).

**Figure 3:**
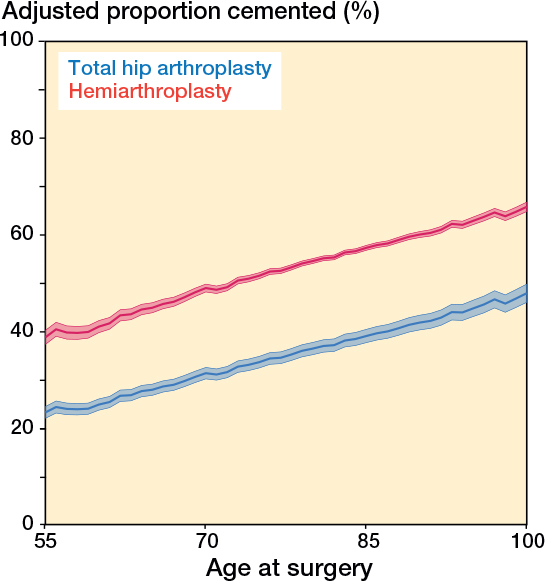
Adjusted proportion of cemented fixation (%; 95% confidence interval) for hip fracture patients by patient age and type of arthroplasty for 2019/20–2021/22. Adjusted by sex, type of arthroplasty, approach, comorbidities, annual hospital hip fracture volume, annual surgeon hip fracture volume, and hospital type.

These trends remained present in the adjusted analysis (Table 2). The OR of cemented fixation for hemiarthroplasty (vs THA) rose from 1.13 (CI 0.99–1.29) at the start of the study period to 2.17 (CI 2.02–2.33) at the end. In the most recent period, 2019/20–2021/22, the OR for hospitals with > 500 hip arthroplasties and over a quarter of cases for fractures was 0.30 (CI 0.27–0.34) and for high volume surgeons (≥ 50 hips) with an above-median fracture volume (≥ 16) the OR was 0.80 (CI 0.75–0.84). Cementing was more common in teaching hospitals, OR 1.16 (CI 1.10–1.22). The marginal proportion of cemented fixation increased with patient age (Figure 3). From 2019/20 to 2021/22, the adjusted proportion increased from around 24% at age 55 to about 39% at age 85 for THA and from 39% to 57% for hemiarthroplasty (Figure 3).

**Table 2:** Adjusted odds ratios (OR; 95% confidence interval) of the association between surgical characteristics and fixation status for patients undergoing arthroplasty for a hip fracture by period

Factor	2012/13–2015/16	2016/17–2018/19	2019/20–2021/22
Age (by year)	1.006 (1.001–1.010)	1.020 (1.017–1.023)	1.023 (1.020–1.026)
Male (vs female)	0.97 (0.89–1.05)	0.91 (0.86–0.97)	0.79 (0.75–0.83)
Comorbidities	0.97 (0.88–1.06)	0.99 (0.93–1.06)	1.19 (1.12–1.26)
Type of arthroplasty			
Total hip arthroplasty	ref.	ref.	ref.
Hemiarthroplasty	1.13 (0.99–1.29)	1.91 (1.75–2.09)	2.17 (2.02–2.33)
Approach			
Lateral/posterior	ref.	ref.	ref.
Anterior	0.86 (0.70–1.07)	1.03 (0.92–1.14)	0.61 (0.56–0.67)
Annual hospital hip arthroplasty volume			
< 500 (< 25% fracture)	ref.	ref.	ref.
< 500 (≥ 25% fracture)	1.50 (1.34–1.69)	0.94 (0.88–1.00)	0.84 (0.79–0.90)
≥ 500 (< 25% fracture)	1.26 (1.10–1.44)	0.67 (0.62–0.72)	0.82 (0.76–0.87)
≥ 500 (≥ 25% fracture)	0.43 (0.27–0.68)	0.32 (0.25–0.41)	0.30 (0.27–0.34)
Hospital type			
Community hospital	ref.	ref.	ref.
Teaching hospital	1.54 (1.41–1.68)	1.32 (1.24–1.40)	1.16 (1.10–1.22)
Annual surgeon hip arthroplasty volume			
< 50			ref.
≥ 50 (< 16 fractures)			0.99 (0.93–1.05)
≥ 50 (≥ 16 fractures)			0.80 (0.75–0.84)

Surgeon information only fully available for 2019/20–2021/22; all estimates are mutually adjusted for the other variables in the table

**Figure 4:**
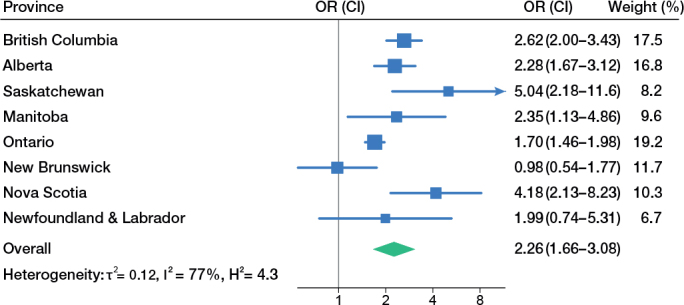
Odds ratios (OR; 95% confidence interval) of the association between hemiarthroplasty (vs total hip arthroplasty) and cemented fixation by province in 2021/22 with random effects meta-estimate.

**Figure S6:**
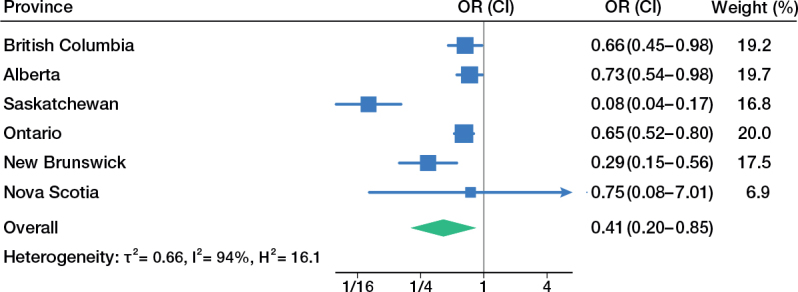
Odds ratios (OR; 95% confidence interval) of the association between anterior approach (vs posterior/lateral approach) and cemented fixation by province in 2021/22 with random effects meta-estimate.

Except for differences in patient age, sex, and comorbidity, there was a large amount of heterogeneity in the estimates for all provinces (Table S3, see Appendix). For example, when looking at the effect of type of surgery (hemiarthroplasty vs THA), the I^2^ was 77% and τ^2^ was 0.12, even though 7 of 9 provinces recorded a statistically significant increase in cemented fixation for hemiarthroplasties in 2021/22. This varied from an OR of 1.7 (Ontario) to 5.0 (Saskatchewan; Figure 4). The effect of the anterior approach is dominated by 1 or 2 provinces and is highly variable across the country (I^2^ 94%; τ^2^ 0.66; Figure S6, see Appendix).

## Discussion

We aimed to identify which factors were associated with a higher likelihood of cemented fixation in hip arthroplasty for hip fracture. We found that the use of cemented fixation increased in almost all groups across Canada between 2012 and 2022, despite interprovincial heterogeneity. Overall, cement use was more prevalent in hemiarthroplasty, older patients, females, and patients with comorbidity. We further found that surgeons and hospitals with the highest hip fracture arthroplasty practice were the least likely to cement, despite the benefits of cemented fixation for all surgeons [6,22].

Healthcare in Canada is organized by province; the large interprovincial heterogeneity may be due to historical differences in training, practice, and publicly funded implant contracts. The increase in each province is probably driven by practice changes of individual surgeons, while each province has different mechanisms to monitor and advise on evidence-based best practices. Regardless of these differences and the interprovincial heterogeneity, the broad-based conclusions hold across Canada, e.g., the higher prevalence of cement use in hemiarthroplasty.

The lower use of cemented fixation with the anterior approach could be due to several factors. The direct anterior approach (DAA) has become more prevalent relatively recently [23] and is more frequently performed by surgeons who have spent less time in practice [24]. As femoral fixation has historically been trending cementless in North America [25,26], more recently trained surgeons may have had less relevant exposure to cementing techniques during their residency and fellowships. These 2 factors combined could make surgeons using the anterior approach less likely to be trained in cemented fixation and less prone to change their practice to use it.

Because cemented femoral fixation has been commonplace outside of North America [12,25,26], the literature on which patients tend to receive cemented femoral fixation is sparse. A German study of 56,000 hip fracture arthroplasties did note that cemented fixation was more common in hemiarthroplasty than THA [15], as we found in Canada. The proportion of cemented hemiarthroplasty cases remained stable (around 50%) in the United States between 2009 and 2017 [27] and increased slightly between 2013 and 2021 (42% to 49% for hemiarthroplasty and 17% to 19% for THA) [12], unlike the large increase we noted in Canada during the same period. Although cemented fixation is universally recommended due to the lower risk of periprosthetic fracture and early revision, a recent review from the United States recommended cemented fixation in patients ≥ 70 years old, particularly for women [28]. The same review outlined several other reasons to opt for cemented fixation, such as Dorr type C, osteoporosis, fragility fractures, and a lack of intraoperative broach stability [28]. Some of these factors are more common in older patients, and our increasing cementing prevalence with patient age may be explained by surgeons considering these same factors in their choice of fixation. A combined analysis of Norwegian and Swedish registries noted that the risk of reoperation with cementless stems increased with age [29].

Although the proportion of cemented fixation increased over time, the overall percentage remains low compared with most other countries. This may be related to barriers to cement usage, such as a lack of training and pre-existing implant contracts. There may be a lack of knowledge on the benefits and safety of cemented fixation as data from Canadian and American registries emerged only recently. Surgeons may have felt their experience with cementless fixation offset any increased revision risk and opted against using cement for their procedures. Our results on surgeon volume and training suggest these factors may be why the use of cemented fixation is relatively low in Canada. It is encouraging to see the increased use of cemented fixation in teaching hospitals as skills learned during training are more likely to be carried into practice. Our analysis found that older and female patients were more likely to have cemented fixation, suggesting surgeons take bone condition into account in their decision on fixation, as sex and age are important predictors of bone quality.

The observations from this study have the potential to be incorporated into provincial quality improvement initiatives, as these initiatives have been proven to work elsewhere. For example, a Norwegian study showed that a quality program to increase the use of cemented fixation improves patient care [30].

### Strengths

A major strength of this study is the use of high-quality datasets with > 99% completeness in the provinces we included in our analysis. Although it is possible that some items are miscoded, this data is entered by well-trained dedicated professionals with clearly defined instructions [19,20], which minimizes the number of mistakes. As all variables are assessed at a single time point, there is no loss to follow-up and the risk of measurement error is minimal.

### Limitations

Although we adjusted our analysis for many relevant variables there were some factors we could not account for that may have biased our results, in particular patient BMI and surgeon training/familiarity with cemented fixation. The risk of bone cement implantation syndrome or cardiopulmonary adverse outcomes due to bone cement is small in hip fracture arthroplasty and does not affect mortality risk after the first postoperative week, while evidence during the first week is mixed [11,31]. However, the use of bone cement requires adequate training. We were unable to ascertain the level of familiarity and training surgeons had with cemented fixation. The procedure codes in the DAD do not distinguish between the lateral and posterior approach, which precluded analyzing these approaches separately. We also could not ascertain which component in THA was cemented. As cemented fixation is recommended to avoid periprosthetic fracture in the femur, only reverse hybrid THA (cementless femoral stem, cemented acetabular shell) would be misclassified. The use of reverse hybrid fixation is relatively rare, so we do not think this misclassification would have a major impact on our results or conclusions.

Because we lack information on some of the important factors listed above, our results may be biased by residual confounding. We also did not perform a formal causal diagram analysis, but it is possible that the results for certain variables are biased due to adjustment for colliders and or mediators.

### Conclusion

The use of cemented fixation in hip fracture arthroplasty has increased across Canada over the last decade. However, high-volume surgeons and hospitals that treat more hip fracture patients are less likely to cement. The proportion of cemented fixation increases linearly with patient age, and cement use is more common in hemiarthroplasty than THA. Cemented fixation is more common in teaching hospitals, providing optimism that this will support its increasing use.

## Notes

CHR: conceptualization, methodology, software, formal analysis, resources, writing—original draft, supervision, project administration, funding acquisition. CP: methodology, software, formal analysis, data curation, writing—review and editing, visualization. GCAW: conceptualization, writing—review and editing, supervision, funding acquisition. ERB: conceptualization, resources, writing—review and editing, supervision, funding acquisition

Parts of this material are based on data and information provided by the Canadian Institute for Health Information. However, the analyses, conclusions, opinions, and statements expressed herein are those of the authors and not necessarily those of the Canadian Institute for Health Information.

Handling co-editors: Cecilia Rogmark and Jonas Ranstam

*Acta* thanks Torbjørn Berge Kristensen and Inari Laaksonen for help with peer review of this manuscript.

## Appendix

**Table S1:** Number (%) of hip fracture patients 55 years or older with comorbidity by fixation status and type of arthroplasty

	Total hip arthroplasty		Hemiarthroplasty	
	cementless n = 6,338	cemented n = 2,791	cementless n = 28,850	cemented n = 27,844
Comorbidity	827 (13)	572 (21)	6,760 (23)	6,532 (24)
Chronic pulmonary disease	86 (1.4)	56 (2.0)	730 (2.5)	674 (2.4)
Dementia	175 (2.8)	157 (5.6)	2,096 (7.3)	1,874 (6.7)
Rheumatological disease	16 (0.3)	11 (0.4)	30 (0.1)	29 (0.1)
Mild liver disease	11 (0.2)	7 (0.3)	54 (0.2)	54 (0.2)
Diabetes with organ failure	408 (6.4)	224 (8.0)	2,905 (10)	2,848 (10)
Renal disease	29 (0.5)	26 (0.9)	234 (0.8)	268 (1.0)
Moderate or severe liver disease	6 (0.1)	<5	20 (0.1)	23 (0.1)
HIV infection	0 (0.0)	0 (0.0)	<5	<5
Congestive heart failure	81 (1.3)	71 (2.5)	829 (2.9)	800 (2.9)
Primary cancer	98 (1.5)	92 (3.3)	732 (2.5)	785 (2.8)
Metastatic cancer	24 (0.4)	37 (1.3)	275 (1.0)	292 (1.0)

**Table S2:** Cementing proportion (%) over time by province by fiscal years from April 1 to March 31

	2012/13	2013/14	2014/15	2015/16	2016/17	2017/18	2018/19	2019/20	2020/21	2021/22
British Columbia (BC)	52.5	56.6	57.4	49.6	52.4	57.1	57.5	60.8	60.5	63.4
Alberta (AB)	56.7	60.4	49.2	34.6	36.3	36.2	36.8	43.2	53.4	49.3
Saskatchewan (SK)	25.5	19.2	15.2	36.0	33.3	54.5	51.7	52.2	51.7	52.6
Manitoba (MB)	6.3	3.4	12.8	10.8	10.8	11.2	17.0	20.0	32.7	35.8
Ontario (ON)	18.6	18.5	24.4	30.5	31.7	38.6	42.0	48.9	54.0	57.1
New Brunswick (NB)	43.8	22.4	26.2	30.6	35.1	27.0	32.4	29.3	31.5	35.3
Nova Scotia (NS)	40.3	30.8	33.8	56.4	59.4	61.1	67.3	72.6	77.4	83.4
Prince Edward Island (PE)	41.5	42.2	24.3	83.0	82.9	80.9	87.9	91.5	96.7	97.4
Newfoundland & Labrador (NF)	67.3	70.4	73.5	84.0	85.5	76.8	79.6	78.6	83.5	86.6
Overall	32.0	32.5	35.8	36.4	37.6	42.7	45.4	50.2	55.1	57.3

**Table S3:** Adjusted odds ratios (OR; 95% confidence interval) of the association between fixation status and, demographic and clinical characteristics for patients undergoing hip fracture treatment for the year 2021/22 by province with a random effects meta-estimate and I^2^.

	BC	AB	SK	MB	ON	NB	NS	NF	Overall (estimate)	I^2^
Age										
55–64	ref.	ref.	ref.	ref.	ref.	ref.	ref.	ref.	ref.	ref.
65–79	0.90 (0.67–1.21)	1.01 (0.70–1.44)	0.44 (0.18–1.07)	1.60 (0.60–4.22)	0.78 (0.66–0.92)	0.89 (0.45–1.73)	1.62 (0.79–3.30)	1.19 (0.42–3.32)	0.89 (0.75–1.05)	16.9
≥80	1.19 (0.88–1.60)	1.49 (1.02–2.18)	0.69 (0.28–1.70)	1.63 (0.61–4.37)	1.20 (1.02–1.42)	1.64 (0.87–3.10)	2.74 (1.32–5.69)	3.02 (1.06–8.63)	1.33 (1.13–1.56)	13.5
Male (vs. Female)	0.81 (0.66–0.99)	0.84 (0.67–1.07)	0.77 (0.47–1.29)	0.74 (0.47–1.17)	0.69 (0.61–0.78)	0.64 (0.39–1.04)	0.60 (0.35–1.04)	1.38 (0.54–3.57)	0.74 (0.67–0.83)	10.7
Comorbidities	1.23 (0.96–1.57)	1.02 (0.77–1.36)	0.69 (0.34–1.39)	1.27 (0.75–2.16)	1.20 (1.05–1.39)	1.07 (0.55–2.09)	2.00 (0.84–4.78)	1.31 (0.41–4.23)	1.18 (1.06–1.31)	0.0
Type of operation										
Total hip arthroplasty	ref.	ref.	ref.	ref.	ref.	ref.	ref.	ref.	ref.	ref.
Hemiarthroplasty	2.62 (2.00–3.43)	2.28 (1.67–3.12)	5.04 (2.18–11.63)	2.35 (1.13–4.86)	1.70 (1.46–1.98)	0.98 (0.54–1.77)	4.18 (2.13–8.23)	1.99 (0.74–5.31)	2.26 (1.66–3.08)	76.7
Approach										
Lateral/Posterior	ref.	ref.	ref.	ref.	ref.	ref.	ref.	ref.	ref.	ref.
Anterior	0.66 (0.45–0.98)	0.73 (0.54–0.98)	0.08 (0.04–0.17)		0.65 (0.52–0.80)	0.29 (0.15–0.56)	0.75 (0.08–7.01)		0.41 (0.20–0.85)	93.8
Annual hospital hip arthroplasty volume										
<500 (<25% fracture)	ref.	ref.	ref.	ref.	ref.	ref.	ref.	ref.	ref.	ref.
<500 (≥25% fracture)	1.77 (1.38–2.28)	1.16 (0.80–1.67)		0.65 (0.28–1.50)	0.71 (0.60–0.83)	0.07 (0.01–0.54)	0.70 (0.39–1.26)		0.86 (0.54–1.36)	87.7
≥500 (<25% fracture)	0.22 (0.16–0.32)	0.54 (0.38–0.76)		2.22 (1.05–4.72)	1.39 (1.15–1.68)				0.76 (0.28–2.05)	97.0
≥500 (≥25% fracture)		0.32 (0.23–0.44)		0.25 (0.11–0.57)					0.31 (0.23–0.41)	0.0
Hospital type										
Teaching hospital	ref.	ref.	ref.	ref.	ref.	ref.	ref.	ref.	ref.	ref.
Community hospital	0.91 (0.73–1.15)	0.85 (0.65–1.12)	0.93 (0.51–1.69)		1.63 (1.41–1.87)	0.08 (0.04–0.18)			0.65 (0.25–1.73)	98.3
Annual surgeon hip arthroplasty volume										
<50	ref.	ref.	ref.	ref.	ref.	ref.	ref.	ref.	ref.	ref.
≥50 (<16 fractures)	0.63 (0.50–0.80)	0.73 (0.51–1.05)	0.94 (0.50–1.76)	2.46 (1.05–5.78)	0.92 (0.77–1.11)	1.09 (0.56–2.11)	1.38 (0.68–2.79)		0.92 (0.71–1.20)	64.7
≥50 (≥16 fractures)	0.96 (0.75–1.22)	0.71 (0.55–0.91)	0.67 (0.34–1.34)	0.77 (0.46–1.29)	0.67 (0.59–0.76)	0.66 (0.29–1.47)	0.50 (0.28–0.90)		0.73 (0.62–0.85)	38.9

All estimates are mutually adjusted for the other variables in the table.
